# Detection of high-risk human papillomavirus (HPV) by the novel AmpFire isothermal HPV assay among pregnant women in Pemba Island, Tanzania

**DOI:** 10.11604/pamj.2020.37.183.23367

**Published:** 2020-10-27

**Authors:** Naomi Christine Angela Juliana, Mohamed Hamad Juma, Roel Heijmans, Sander Ouburg, Said Mohammed Ali, Aishwarya Singh Chauhan, Amanhi Biobank Pemba, Sunil Sazawal, Servaas Antonie Morré, Saikat Deb, Elena Ambrosino

**Affiliations:** 1 Institute of Public Health Genomics, Department of Genetics and Cell Biology, Research School GROW, Maastricht University, Maastricht, The Netherlands,; 2 Public Health Laboratory, Ivo de Carneri, Chake Chake, Pemba Island, Tanzania,; 3 Laboratory of Immunogenetics, Department of Medical Microbiology and Infection Control, Amsterdam UMC Location AMC, Amsterdam, The Netherlands,; 4 Centre for Public Health Kinetics, New Delhi, India

**Keywords:** HPV infection, pregnancy, AmpFire HPV, isothermal, sub-Saharan Africa, HPV persistence

## Abstract

**Introduction:**

human papillomavirus (HPV) is the most common sexually transmitted virus in the world. Prevalence of infection differs, with highest rates reported in sub-Saharan African, including the country of Tanzania. In pregnancy, the hormonal changes and immune changes seem to facilitate HPV persistence, increasing the cancer risk and the risk of vertical transmission towards the placenta and the fetus. The burden of HPV infection is still high despite multiple screening and detection test available. The AmpFire® HPV assay is a novel nucleic acid isothermal amplification with real-time fluorescence detection assay that can test simultaneously 15 high-risk HPV. This nested cohort study aims to contribute evidence on the prevalence of HPV infection and persistence across two time points among pregnant women in Pemba island, Tanzania.

**Methods:**

vaginal swabs that were previously collected during pregnancy were stored in eNAT buffer (n_1_=385 and n_2_=187) and were tested with AmpFire® screening assay, for simultaneous detection of the HPV 16, 18 and other high-risk HPV genotypes 31, 33, 35, 39, 45, 51, 52, 53, 56, 58, 59, 66 and 68.

**Results:**

the AmpFire® HPV assay detected an 11% and 6% high-risk HPV prevalence at the two time points among pregnant women in Pemba island, consecutively. For the 133 women whose samples were tested at both time points, the persistence rate of high-risk HPV was 64%.

**Conclusion:**

novel isothermal HPV assay, such as the AmpFire®, might be feasible to use in low-income regions.

## Introduction

Genital human papillomavirus (HPV) is the most common sexually-transmitted virus in the world. Prevalence of infection differs among regions, with the highest rates reported in South America and sub-Saharan Africa, including the country of Tanzania [1]. In most cases, vaginal HPV infections are transient and cleared by the body. In other cases, the virus might persist and cause numerous pathologies in the female reproductive system [1]. Such differences might be caused by host genetic factors or by different HPV strains [2]. The presence of high-risk HPV (hrHPV) infection during pregnancy has been associated with various adverse pregnancy-related complications or outcomes, such as vaginal infection, preterm birth (PTB) and preterm pre-labor rupture of membranes [3,4]. In pregnancy, hormonal and immune changes seem to facilitate HPV persistence, therefore increasing the cancer risk and the risk of vertical transmission towards the placenta [5]. Cervical infection with hrHPV has been particularly associated with placental abnormalities and PTB [4,6]. Vertical transmission from HPV infected mothers to their children during pregnancy, labor and delivery has also been reported in multiple studies [6].

Considering the significance of HPV in numerous pathologies, the interest to develop innovative diagnostic methods has grown. To date, there are several molecular diagnostic tests available to detect hrHPV genotypes [7]. Even though the southern blot is the gold standard for HPV genomic analysis, it has low sensitivity and it is time-consuming. In addition, it needs a large amount of purified DNA [7]. Luckily, higher sensitive and specific HPV signal or nucleic acids amplification assays, such as hybrid capture II (Qiagen, Australia), Aptima (Hologic Inc, USA) and Cobas 4800 (Roche Molecular Diagnostics, Switzerland), are currently used in most HPV screening programs [8]. Recently, assays based on the isothermal amplification technique, such as the AmpFire Multiplex HPV assay (Atila BioSystems, Inc., CA, USA), are some of the newer isothermal methods available. Compared to the widely available APTIMA HPV isothermal assay, the real-time fluorescent multiplex nucleic acid amplification AmpFire assay can test more hrHPV genotypes in a single reaction tube. It can distinguish between HPV 16 (cyanine5 fluorophore (CY5™)), HPV 18 (carboxyrhodamine (ROX™)), other hrHPV genotypes (fluorescein amidites (FAM™)): 31, 33, 35, 39, 45, 51, 52, 53, 56, 58, 59, 66 and 68, (hereafter referred to as other hrHPV) and internal control (HEX™). Sequence-specific primers targeting each of the 15 hrHPV genotypes are used in the isothermal amplification system to amplify targeted sequences in the different HPV genotype regions.

AmpFire HPV assay was Conformité Européenne (CE, European community) marked in 2017 and received Chinese food and drug administration approval in December 2015. For self-collected vaginal samples, the AmpFire test has shown similar specificity (92.9%) and almost comparable sensitivity (92.9%) rates as the Roche Cobas 4800 [9,10]. Besides, the AmpFire assay is expected to be easy to use in low-income settings, since the isothermal method does not require an advanced polymerase chain reaction (PCR) machine, requires almost no sample processing for HPV detection and gives results in a short amount of time (AmpFire HPV screening assay kit user manual). As such, this methodology might prove useful in resources-constrained settings, such as Tanzania, where ideal tests should be affordable and require minimal extra tools and training. Consistent with the association between hrHPV and adverse pregnancy outcomes, HPV detection during pregnancy will contribute towards optimising a better female reproductive and child health care in Tanzania [11,12]. It has been previously suggested that during pregnancy the hormonal and immunological factors lower hrHPV clearance [5,13-15]. However, information on HPV persistence during pregnancy is not well established and data on HPV persistence during pregnancy in sub-Saharan Africa is scant [15-17]. Therefore, this study aims to assess the prevalence and persistence of hrHPV infection in samples collected among pregnant women in Tanzania using the AmpFire HPV assay.

## Methods

**Sample collection:** the vaginal samples were collected in the context of a previously established biobanking effort (AMANHI) with the support of the Bill and Melinda Gates Foundation [18] initiated in 2014 in Pemba island, Tanzania. Pregnant women of more than 8 weeks of Gestational Age (GA) and who gave their informed consent for sample collection were eligible to participate in the biobanking effort [18]. The vaginal swabs were collected at three different time points of their pregnancy and postpartum period (for the present study we have used two of them) in health clinics during antenatal visits and under the supervision of a health worker. The study has received ethical approval from the local Zanzibar Medical Research and Ethics Committee (ZAMREC) [18]. For the analysis of this retrospective study, a total of 572 vaginal swabs from 439 women that were collected between March 2018 and January 2019 were used. Three hundred eighty-five vaginal swabs were collected between 8-19 GA weeks of as the first time point samples and 187 vaginal swabs were collected either at 24-28 GA weeks or 32-36 GA weeks as the second time point samples. In total, 133 women participated in this study at both time points. The vaginal swabs were preserved in 1ml eNAT buffer (Copan Italia, Brescia, Italy) and stored at -20°C. Samples were transported on dry ice to the Netherlands and stored at -24°C until further processing. Vials of samples that had less than 300μl eNAT buffer content were not tested.

**DNA isolation:** DNA was extracted from the collected vaginal swabs with the Chemagen (Perkin-Elmer, Germany) automated DNA extraction machine by using the buccal swab extraction kit according to the manufacturer´s instructions and were afterwards stored at 5°C [19].

**AmpFire HPV screening assay:** the AmpFire HPV screening assay kit (Atila BioSystems, CA, USA) was used in this study following the information in the user manual provided in the kit. The kit includes reaction mix, primer mix, external positive control template and negative control template. Briefly, 12μl reaction mix was combined with 11μl primer mix in a 0.2ml optical 96-well plate. Two μl of processed DNA samples were added to the reaction tube to bring the total volume to 25μl. Real-time PCR was performed using the Applied Biosystems™ 7500 Real-Time PCR system with the isothermal reaction condition set at 60°C while taking fluorescence dye reading at the FAM™/HEX™/CY5™/ROX™ channels once every minute for a total of 75 minutes. The thermocycling software system automatically reports the results of the cycle threshold (Ct) values for each amplification curve in all fluorescence channels. For each sample, an exponential amplification curve in CY5™, ROX™, FAM™ and HEX™ channels indicates the presence of DNA of HPV 16, HPV 18, other hrHPV genotypes and internal control respectively. The lack of exponential amplification curve in the HEX channel was interpreted as an invalid result. The test results were used to determine: hrHPV point prevalence; co-infections of HPV 16 and/or HPV 18 with other hrHPV; persistence and incidence rate of hrHPV during pregnancy.

**Statistical analysis:** dichotomous variables were generated as pregnant women were either negative or positive for hrHPV infection. Persistence of infection was considered when the vaginal samples of the same women tested positive for HPV infection at both timepoints. Chi-square test was used to determine whether the point prevalence changed significantly over time during pregnancy. A p-value of <0.05 indicated a significant difference.

## Results

**Figure 1:**
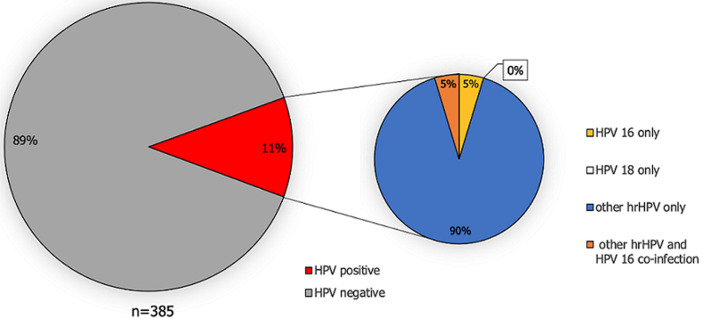
prevalence of HPV infection and high-risk HPV genotype distribution in the first vaginal sample of pregnant women in Tanzania (decimals have been rounded to full numbers)

**Figure 2:**
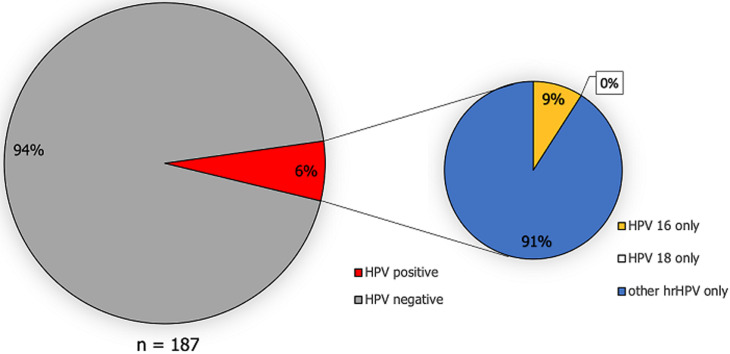
prevalence of HPV infection and high-risk HPV genotype distribution in the second vaginal sample collection among pregnant women in Tanzania (decimals have been rounded to full numbers)

**HPV prevalence:** a total of 11.2% (43/385) of pregnant women tested positive for HPV infection at the first time and 5.9% (11/187) at the second time point (Figure 1, Figure 2). Of the women who tested positive, none of them were infected with HPV genotype 18. However, for HPV 16, 4/385 pregnant women tested positive at the first time point and 1/187 pregnant women at the second time point. The majority of the detected HPV infections were caused by other hrHPV genotypes (Figure 1, Figure 2). Among 133 women who were tested at both time points, the overall hrHPV prevalence was not significantly higher at the first time point (8.3%, 11/133) compared to the second time point (6.8%, 9/133) (Х^2^ = 0.22; *p*-value=0.64).

**Figure 3:**
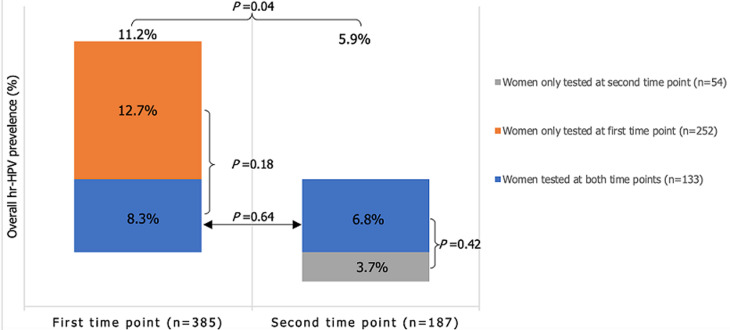
three categorical groups of women tested positive for hrHPV at time point one or time point two; arrows and accolades indicate whether or not the difference in two prevalences are statistically significant; the percentage of infected women in each group at both time points were not significantly different (*p*-value >0.05)

For women tested only once at either time point, the overall hrHPV prevalence was 11.1%, HPV16 prevalence was 1.5% and other hrHPV prevalence was 10.5% (data not shown in the figures). Within this group of women, the overall hrHPV prevalence at the first time point was 12.7% and at the second time point 3.7%. The distribution of the infected women was not symmetrical within both time point (Figure 3). The hrHPV prevalence at the first time point is borderline significant (p-value=0.04) as compared to the second time point. Whilst, the number of infected women tested at both time points compared to the women tested only at first or second time point, were not significantly different at either time point (Figure 3).

**Co-infections:** only at the first time point, the assay detected multiple hrHPV infections in two women. These two women tested positive simultaneously for HPV 16 and other hrHPV genotypes.

**The persistence rate and new incidences:** among the 133 pregnant women tested at both time points, eleven were hrHPV positive at the first time point, and seven remained positive at the second time point. The persistence rate for hrHPV was 63.6% in this subgroup. Among the women who tested negative for HPV at time point one, two acquired a new HPV infection and resulted positive at the second time point. One of the women had a de novo HPV 16 infection and a different women was superinfected with other hrHPV during the course of her pregnancy.

## Discussion

In this cohort of pregnant women in Pemba island Tanzania, the hrHPV point prevalence was between 5.9% and 11.2%. The overall hrHPV positive status was higher in an earlier pregnancy stage compared to a later stage, although the difference was borderline significant. There is no logical biological argument why the hrHPV prevalence should be higher at the start of the pregnancy or why it should clear during pregnancy. Moreover, when the comparison was made between the paired samples, no significant difference was observed between the two time points. Once the study is complete at all time points, a more definite answer can be drawn.

Previous studies showed a higher hrHPV infection rate among pregnant women, compared to non-pregnant ones [20]. Unexpectedly, both hrHPV point prevalence of this study were lower than the previously reported overall prevalence of 20% observed in non-pregnant women across rural and urban areas in mainland Tanzania [21]. This difference can be explained by regional variation and the cultural impact of religion on sexual behavior [21,22].

Compared to other pregnant cohort studies, differences in geographical regions with varying exposures to infection and risk factors might explain the dissimilar HPV prevalence. This might partially explain the broad range in hrHPV prevalence reported: among Ghanaian (21% and 46%), Lithuanian (7-9%), Brazilian (25%), Turkish (15%) and Indian (39%) pregnant women [3,20,23-26]. However, the low overall HPV prevalence observed among pregnant women in Nigeria (5.4%, IgM serology based) [27] and in rural South Africa (5.7%, cytology based) [28] are more in line with the prevalence observed in the current study.

With regard to the persistence of hrHPV, we found a maximum rate of 64% in this cohort. This AmpFire screen HPV assay does not distinguish between hrHPV genotypes other than HPV 16 or HPV 18. Therefore, the exact persistence rate of specific hrHPV genotypes might be lower since now those fall within the same other hrHPV group. In addition, some women might have had triple or even multiple HPV genotype infections. Nevertheless, the persistence rate during pregnancy found in this study is roughly comparable with previous findings among pregnant women in Uganda (50.4% and 71.8%), Brazil (53%) and the Netherlands (58%), but is higher than in human immunodeficiency virus (HIV) positive pregnant women in Spain (46%) [5,13,15,29]. However, it should be noted that the method to determine HPV status, the types of HPV genotypes analyzed, the method to determine the persistence or clearance, the number of primiparous women, the genetic background of participants, their nutritional and health status and the follow-up periods between studies differ and should be taken into account when comparing viral clearance in different populations.

The AmpFire assay was previously compared to the Roche Cobas 4800 HPV assay and showed comparable sensitivity and specificity [10]. Also, when we compared the AmpFire assay with our in-house assay, we observed a 100% concordance between the results (data not shown). Further comparison studies are highly recommended to determine the diagnostic values of this assay compared to others. In this study, we used DNA that had been previously isolated by the validated Chemagen automated technology, as the samples were prior tested for other infections. This approach of using pure DNA might have resulted in an overestimation of the hrHPV prevalences compared to the DNA isolation method from Atila BioSystems, which is simpler, possibly less effective, thus have a higher chance for inhibition of the amplification reaction. The AmpFire HPV assay does not require DNA extraction or purification. Therefore, the simple sample processing procedure might be helpful for HPV screening in clinical diagnostic settings, especially in resource-limited areas.

To our knowledge, this study is the first that investigated the prevalence of HPV in pregnant women in Pemba island, Tanzania. The sample size included in this study is higher than other pregnancy studies conducted in Uganda, Kenya, Nigeria, Ghana, South Africa, Brazil, Lithuania and India.

## Conclusion

Among pregnant women in Pemba island the hrHPV prevalence is rather low, but not negligible and the persistence rate seems to be high. This study highlights the importance of monitoring this viral infection during pregnancy. Therefore, good diagnostics methods for the detection and screening of HPV in pregnancy are essential to manage the burden of infection, as well as further co-infections that might affect the mother, fetus or newborn.

### What is known about this topic

- HPV is the most common sexually transmitted virus in the world, with the highest rates reported in South America and sub-Saharan Africa;
- In pregnancy, hormonal and immune changes seem to facilitate HPV persistence, therefore increasing the cancer risk and the risk of vertical transmission towards the placenta;
- High-risk HPV infection during pregnancy has been associated with various adverse pregnancy-related complications or outcomes, such as vaginal infection, preterm birth and preterm prelabor rupture of membranes.

### What this study adds

- The prevalence of high risk HPV is between 5.9% and 11.2% in pregnant women in Pemba island, Tanzania;
- The persistence rate for high risk HPV during pregnancy was high (63.6%) in this Tanzanian cohort.
